# [Corrigendum] Leptin promotes breast cancer cell migration and invasion via IL-18 expression and secretion

**DOI:** 10.3892/ijo.2026.5864

**Published:** 2026-02-26

**Authors:** Kuangfa Li, Lan Wei, Yunxiu Huang, Yang Wu, Min Su, Xueli Pang, Nian Wang, Feihu Ji, Changli Zhong, Tingmei Chen

Int J Oncol 48: 2479-2487, 2016; DOI: 10.3892/ijo.2016.3483

Following the publication of the above article and an Expression of Concern statement that was issued in light of concerns raised by an interested reader (doi: 10.3892/ijo.2025.5816) regarding potential duplications of data comparing Figs. 1D and 2E, and the apparent re-use of β-actin control data in Fig. 4A and B where different experimental conditions were reported, the authors have now responded to the enquiry posed by the Editorial Office. After consulting their original data, the authors have realized that the data in Fig. 2E for the MDA-MB-231 cell line, and the β-actin blots in Fig. 4B, were chosen incorrectly (it was also noted by the Editorial Office, upon performing an independent analysis of the data in this paper, that, in Fig. 6C, the CD68/Lep. and CD163/CNL.+Lep. data panels appeared to contain an overlapping section of data, such that the data shown in these panels may have been derived from the same original source).

The revised versions of Figs. 2 and 4 (showing the correct data for the MDA-MB-231 cell line in Fig. 2E and the β-actin blots in Fig. 4B) are shown on the next two pages. Furthermore, a revised version of Fig. 6, showing replacement data for the CD68/Lep. data panel, is also shown on the third page. Note that the errors made in assembling these figures did not affect the overall results and conclusions reported in the paper. The authors are grateful to the Editor of *International Journal of Oncology* for granting them the opportunity to publish this corrigendum, and all the authors agree with its publication; furthermore, they apologize to the readership of the journal for any inconvenience caused.

## Figures and Tables

**Figure 2:**
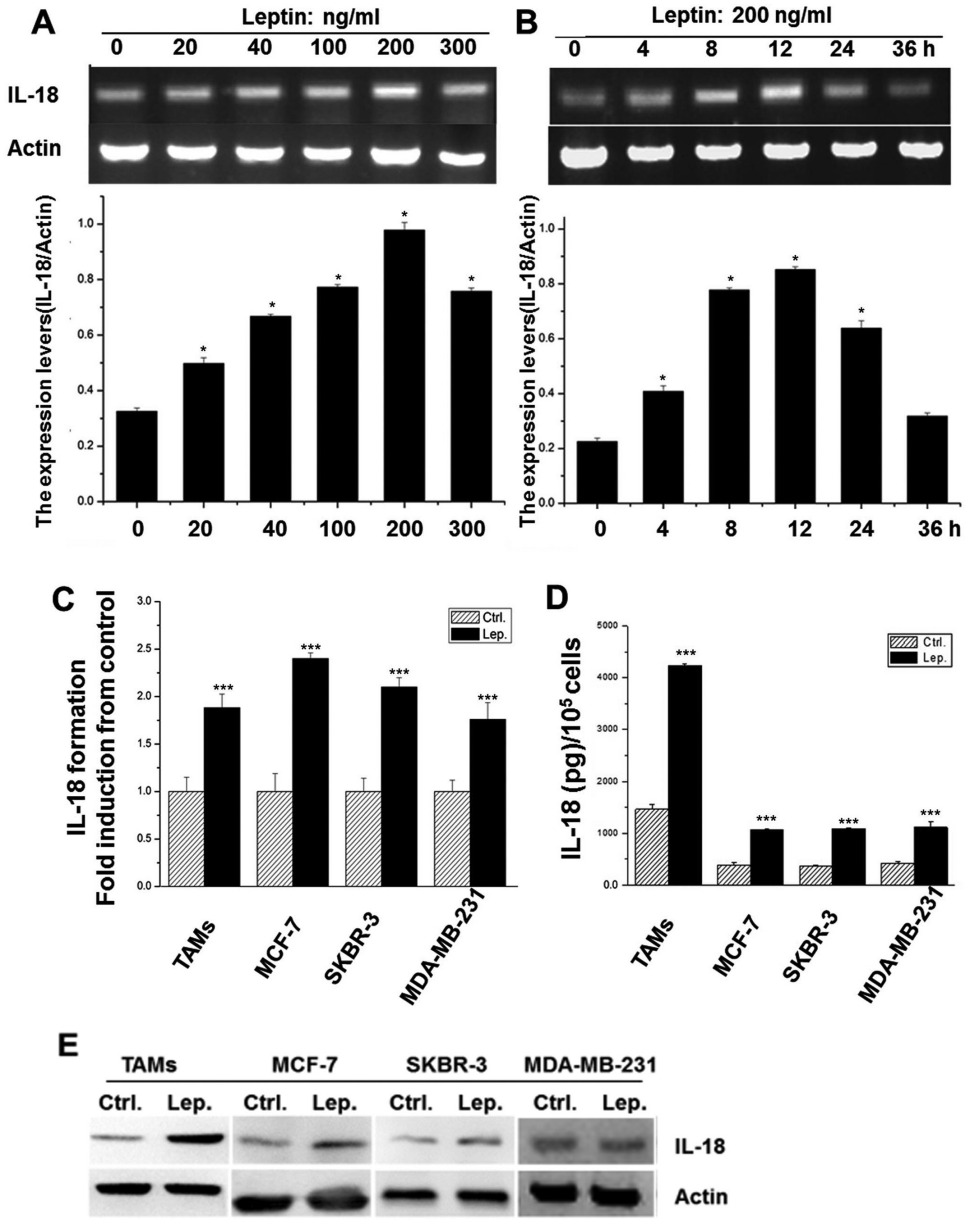
Leptin-stimulated IL-18 mRNA, protein expression and secretion. (A) TAMs were incubated with leptin at the indicated concentrations for 24 h. IL-18 mRNA expression by RT-PCR, Lower part: normalized mRNA levels determined by densitometry. (B) TAMs were treated with leptin (200 ng/ml) for indicated periods of time. IL-18 expression was analyzed as in (A). Lower part: normalized mRNA levels. (C) Leptin-induced IL-18 mRNA expression was confirmed by qRT-PCR. (D) IL-18 secretion into the supernatant was measured by ELISA in TAMs, MCF-7, SKBR-3 and MDA-MB-231. (E) Leptin-induced IL-18 protein expression was confirmed by WB in TAMs, MCF-7, SKBR-3 and MDA-MB-231. Results are shown as fold-changes compared with the control, *P<0.05, ^***^P<0.01 compared to untreated (n=3 experiments).

**Figure 4:**
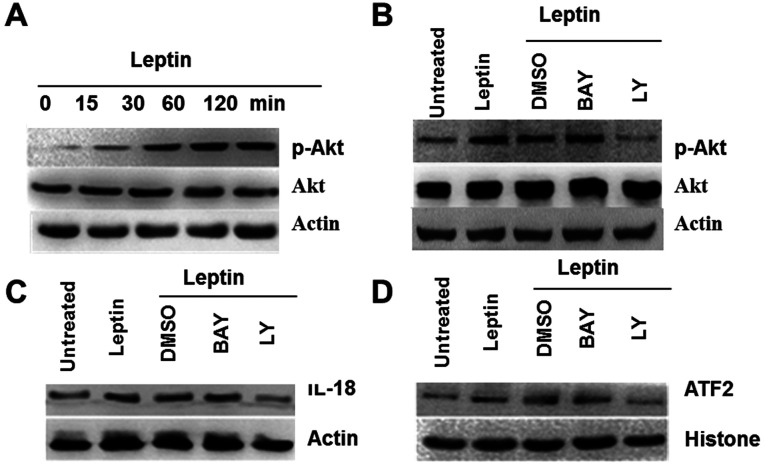
Leptin induces IL-18 expression via PI3K-Akt signaling pathway in MCF-7. (A) Leptin activated Akt. MCF-7 cells were incubated with leptin for up to 2 h. The peak level of p-AKT expression was observed at 1 h. (B) LY depressed IL-18 protein expression in MCF-7 treated by leptin. TAMs were pretreated with NF-κB inhibitor BAY (50 μmol/l) and PI3K inhibitor LY (10 μmol/l), DMSO served as controls. (C) Treatment with BAY blocked IL-18 induction of NF-κB activation. MCF-7 cells were treated with LY (10 μM in DMSO for 1 h) before leptin addition (200 ng/ml for 30 min). (D) LY inhibitor the transcription factor ATF2 of IL-18 in nuclear protein of MCF-7, histone served as a loading control. (n=3 experiments).

**Figure 6:**
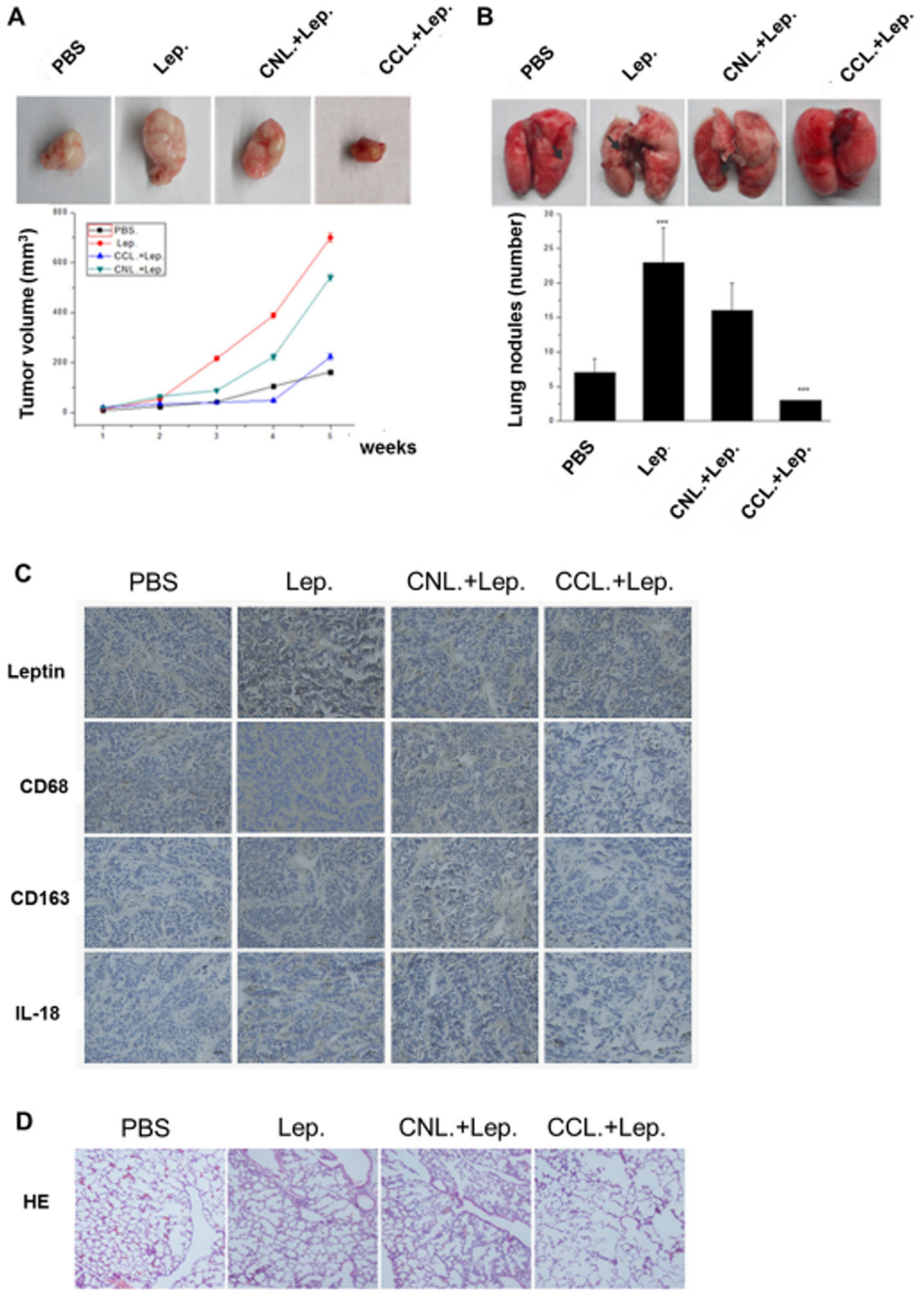
Effect of leptin and macrophages on organ metastasis in the nude mouse xenograft model. (A) Groups of female nude mice (n=5) were injected 1×10^6^ MCF-7 breast cancer cells in mammary fat pad and 15 days after tumor inoculation, intraperitoneal injections with PBS, leptin, macrophage depletion clophosome-clodronate liposomes (CCL) or control neutral liposome (CNL) (0.1 ml) on days 10, 15, 20 and 25, the size of tumors was determined weekly. (B) The number of lung metastases was plotted and representative images of the lungs are shown. For lung metastases, ^***^P<0.01 compared to untreated or controls. (C) Immunohistochemical staining of leptin, IL-18, CD68 and CD163 of tumor tissue sections. (D) H&E staining of lung metastasis in mice.

